# The influence of valence and decision difficulty on self-referential processing

**DOI:** 10.3389/fnhum.2013.00046

**Published:** 2013-02-28

**Authors:** Harma Meffert, Laura Blanken, Karina S. Blair, Stuart F. White, James R. Blair

**Affiliations:** ^1^Section of Affective and Cognitive Neuroscience, National Institutes of HealthBethesda, MD, USA; ^2^Department of Child and Adolescent Psychiatry/Psychology, Erasmus MC - Sophia Children's HospitalRotterdam, Netherlands; ^3^National Institute of Mental Health, National Institutes of HealthBethesda, MD, USA

**Keywords:** fMRI, self-referential processing, decision making, emotion, affect

## Abstract

Self-referential processing is defined as the process by which a person becomes aware that specific contents are related to his or her own self. Cortical midline structures (CMS), such as dorsal and medial prefrontal cortex, and regions such as inferior frontal cortex, insula, and temporal pole have been implicated in self-referential processing. However, the specific contribution of each of these areas is still largely unknown. More particularly, not many studies have examined the influence of valence and decision making difficulty on regions involved in self-referential processing. In this study, participants evaluated how well personality traits, differing in valence and decision difficulty, described themselves or the current US President. In line with predictions, ventral, rostral, and dorsal parts of medial prefrontal cortex showed greater activity when participants judged traits about themselves relative to judging traits about the current US President. However, none of these regions showed significant modulation by trait valence. Increasing trait decision difficulty was associated with increased activity within dorsal medial prefrontal cortex and bilateral anterior insula. However, there was very minimal overlap (6/119 voxels, i.e., 5%) of the regions of dorsal medial prefrontal cortex implicated in self-referential processing and those implicated in trait decision difficulty. The results are interpreted within current accounts of self-referential processing.

## Introduction

Self-referential processing is defined as the process by which a person becomes aware that specific contents are related to his or her own self (Northoff et al., 2011). Regions implicated in what has been termed conceptual self-referential processing (Powell et al., 2010) include cortical midline structures [CMS; rostral/ventral medial prefrontal cortex (r/vMPFC), posterior cingulate cortex (PCC)/precuneus and, to a lesser extent, dorsal medial frontal cortex (dMPFC)] (Amodio and Frith, 2006; van der Meer et al., 2010; Northoff et al., 2011) as well as inferior frontal cortex, insula and the temporal poles (van der Meer et al., 2010; Northoff et al., 2011). Within the CMS, it appears that r/vMPFC shows the strongest differentiation between self and other processing (van der Meer et al., 2010). Indeed, there have been suggestions that rMPFC is particularly involved in self-referential processing while more dorsal aspects of the MPFC may be more important for other-referential thinking (Mitchell et al., 2006). It is important to remember, however, that CMS have also been implicated in emotional processing (Etkin et al., 2011; Lindquist et al., 2012). Self-referential tasks frequently involve judgments regarding personality traits with high affect. Moreover, the tasks used to study self-referential processing (e.g., asking someone to indicate whether a personality trait applies to them) have a significant decision making component. The dMPFC in particular has been implicated in several specific computational processes related to decision making; e.g., conflict monitoring (Botvinick et al., 2004) and the representation of response-outcome combinations (Alexander and Brown, 2011).

Varying not only the referential target (self or other) but also the valence and the difficulty of decision making can help determine whether regions implicated in self-referential processing are recruited mainly because of their specific role in self-referential processing or because of more general roles in affect and/or decision making. Some previous work has examined the impact of affect on self-referential processing of personality traits (Fossati et al., 2003; Phan et al., 2004; Moran et al., 2006; Gutchess et al., 2007; Northoff et al., 2009). Most of this work has concluded that rMPFC/CMS more generally is not modulated by stimulus valence during self-referential processing (Fossati et al., 2003; Phan et al., 2004; Moran et al., 2006; Gutchess et al., 2007; Northoff et al., 2009). However, Moran et al. (2006) reported that a region within subgenual cingulate cortex showed greater activity in response to positive relative to negative items—though only when they were deemed highly self-relevant. Moreover, it should be noted that both Enzi et al. (2009) and de Greck et al. (2008) found notable overlap between regions engaged in self-referential processing and those related to reward-related processing in the context of a gambling task.

To our knowledge, no previous work has examined the impact of self-referential decision difficulty on activity within regions implicated in self-referential processing. This is despite influential criticism of the self-referential literature, which attributes much of the activity within MPFC to inferential/decision making processes rather than self-referential processing *per se* (Legrand and Ruby, 2009). Indeed, as noted by Northoff et al. (2011), the “association of … task-specific requirements with the midline structures during presentation of self- and non-self-specific stimuli remains to be investigated.” Decision/task difficulty has been manipulated in a variety of tasks; e.g., fluid reasoning (Kalbfleisch et al., 2007), time perception (Livesey et al., 2007), n-back task (e.g., Lythe et al., 2012), and Stroop/Stroop-like tasks (see for example Fellows and Farah, 2005; Grinband et al., 2011; Jasinska et al., 2012; Sheth et al., 2012). This literature has relatively consistently shown that dorsomedial frontal cortex increases activity as decision/task difficulty increases (Paus et al., 1998; see Kalbfleisch et al., 2007; Livesey et al., 2007; Grinband et al., 2011; Sheth et al., 2012). This is consistent with many theories of cognitive control that have stressed the importance of dmPFC, either as a performance monitor or as a hub implementing cognitive control (Shackman et al., 2011). Interestingly, Alexander and Brown have more specifically modeled the role of dmPFC in decision-making (the Predicted Response-Outcome (PRO) model; Alexander and Brown, 2011). According to this model, the dmPFC maps the prediction of various response-outcomes combinations: “This suggests that mPFC may signal a greater number of predicted or actual responses or outcomes instead of a response conflict *per se*, as found previously with neurophysiological studies. The PRO model simulates these findings […] which yields an overall net increase in signals predicting the correspondingly greater number of motor responses” (Alexander and Brown, 2011, p1341). One of the predictions of this model is a stronger aggregate signal with increased number of response options. This prediction was confirmed by Marsh et al. (2007) who showed that dmPFC increased activity as a function of the number of differentially reward options to choose between in a decision making paradigm increased.

Following previous work (Craik et al., 1999; Kelley et al., 2002), we examined participants' BOLD responses when they evaluated how well a personality trait described them or the current US president. To minimize the effect of perceptual processes on self-referential processes, the same personality traits were used for the self and other condition. Both positive and negative traits [as indexed respectively by high and low likeableness scores (Anderson, 1968)] were examined, allowing us to determine the impact of valence on regions implicated in self-referential processing. Decision difficulty was manipulated by having traits that were either highly positive or negative (so called high intensity items such as kind and cowardly) or moderately positive or negative (so called low intensity items such as deliberate and listless). We hypothesized that judgments of more extreme items are easier than judgments of less extreme items as the participant is more likely to have a prepotent judgment associated with such an item; i.e., there would be less response options associated with the item). This hypothesis was supported by our pilot data; subjects demonstrated more response variability when judging low intensity items and were slower when responding to low intensity items. On the basis of Moran and colleagues (2006), we predicted that if we were to see valence modulating activity during self/other-referential judgments, it would be within subgenual vMPFC. On the basis of recent modeling showing that increased potential response options are associated with greater dMPFC activity (Alexander and Brown, 2011), we predicted that low intensity traits would be associated with greater dMPFC activity than high intensity traits. Moreover, we predicted that if decision making processes underlie the medial frontal cortical responses implicated in self-referential processing, then the impact of referential target (self vs. other) and decision difficulty (low intensity vs. high intensity) should show considerable overlap. Alternatively, if they do not, then the medial frontal cortical responses to referential target and decision difficulty should dissociate. The current study tests these predictions.

## Methods

### Study 1: referential processing

#### Participants

Twenty healthy adult volunteers (55% male; average age: 25.05 ± 3.90) were recruited from the community through newspaper ads and fliers. Participants were in good physical health as confirmed by a complete physical exam, with no history of any psychiatric illness as assessed by the DSM-IV (American Psychiatric Association, 1994) criteria based on the Structural Clinical Interview for DSM-IV Axis I disorders (SCID, First et al., 1997). All participants gave written informed consent to participate in the study, which was approved by the National Institute of Mental Health Institutional Review Board. IQ was assessed with the Wechsler Abbreviated Scale of Intelligence (two-subtest form, Wechsler, 1999) and demonstrated an average IQ of 118.79 (*SD* = 7.70).

#### Stimuli and behavioral procedure

Stimuli were selected from Anderson's list of personality traits (Anderson, 1968). This list has often been used in self-referential paradigms (see for example Craik et al., 1999; Kelley et al., 2002). Positive and negative words were selected (e.g., “capable” and “mean”) of high and low intensity as indexed by the likeableness scores (ranging from 0 = “least favorable or desirable” to 6 = “most favorable or desirable,” Anderson, 1968) in the following manner: Words on the extreme ends of the likeableness scale were defined as high intense items, words with moderate likeableness scores were defined as low intense items, words with low likeableness scores were defined as negative words and words with high likeableness scores were defined as positive words. We sampled words evenly throughout the list and only used words with a standard deviation of the likeableness score below 1.5 (Table A1). To avoid possible perceptual and categorical confounds, the same stimuli were used for both the self and other condition. In addition, all four stimulus categories were matched on word length and a second study (see “Personality Traits Questionnaire”) was performed in which all personality traits were rated on valence, arousal, familiarity, and imageability. Three separate ANOVAs showed that words within each condition did differ on likeableness [*F*_(3, 60)_ = 289.266, *p* < 0.001], but not on word length [*F*_(3, 60)_ = 0.545, *p* = 0.654] or meaningfulness [*F*_(3, 60)_ = 1.489, *p* = 0.227]. *Post-hoc* tests indicated that the likeableness scores for every condition differed significantly from the other three. High intensity negative words had a likeableness score of 1.15 (±3.64), low intensity negative words of 2.13 (±3.07), high intensity positive words of 4.79 (±2.67) and low intensity positive words of 3.67 (±5.28). Statements were either self-referential (e.g., “I am capable”) or other-referential (e.g., “… is capable”). Participants were asked to judge, via button press, the extent to which they agreed with the statement presented to them. Following previous work (e.g., Kelley et al., 2002), the subject for “judging others” was the head of state at the time of scanning.

Each trial involved the presentation of a statement for 3600 ms and the participant had to indicate the degree to which they agreed (completely disagree, disagree, agree, completely agree) with that statement during this time (otherwise it was counted as a missed trial). Trials were followed by a 500 ms fixation cross. The task consisted of four runs. Each run consisted of 100 trials: eight of each of the eight stimulus conditions and 36 fixation trials to provide a baseline. Run order and trial order within runs were randomized across participants.

#### MRI data analysis

***MRI parameters.*** Participants were scanned during task performance using a 3-T GE Signa scanner (GE Healthcare, Chalfont St Giles, England). A total of 133 functional images per run were taken with a gradient echo planar imaging (EPI) sequence (repetition time = 2900 ms; echo time = 27 ms; 64_64 matrix; 90° flip angle; 24-cm field of view). A repetition time of 2900 ms was the shortest TR that allowed us full brain coverage with our chosen voxel size. Whole brain coverage was obtained with 46 axial slices (thickness, 3 mm; in-plane resolution, 3.75 × 3.75 mm). A high-resolution anatomical scan (3-dimensional spoiled gradient recalled acquisition in a steady state; repetition time = 7 ms; echo time = 2.984 ms; 24-cm field of view; 12° flip angle; 128 axial slices; thickness, 1.2 mm; 256 × 192 matrix) in register with the EPI data set was obtained covering the whole brain.

***Imaging data preprocessing.*** Imaging data were preprocessed and analyzed in AFNI (Cox, 1996). At the individual level, functional images from the first 5 repetitions were collected before equilibrium magnetization was reached and were discarded. Functional images from the 4 time series were motion corrected and spatially smoothed with a 6-mm full-width half-maximum gaussian filter. The time series were normalized by dividing the signal intensity of a voxel at each point by the mean signal intensity of that voxel for each run and multiplying the result by 100. Resultant regression coefficients represented a percentage of signal change from the mean.

Following this, the following eight regressors were generated: self-referential high intensity negative traits (Self-High-Neg), self-referential low intensity negative traits (Self-Low-Neg), self-referential high intensity positive traits (Self-High-Pos), self-referential low intensity positive traits (Self-Low-Pos), other-referential high intensity negative traits (Other-High-Neg), other-referential low intensity negative traits (Other-Low-Neg), other-referential high intensity positive traits (Other-High-Pos), and other-referential low intensity positive traits (Other-Low-Pos). These indicator functions were then convolved with a gammavariate hemodynamic response function to account for the slow hemodynamic response and used as regressors for our first-level analyses. Linear regression modeling was performed using the eight regressors described above plus regressors to model a first-order baseline drift function. This produced a β-coefficient and associated *t* statistic for each voxel and regressor. The participants' anatomical scans were individually registered to the Talairach and Tournoux atlas (Talairach and Tournoux, 1988). The individuals' functional EPI data were then registered to their Talairach anatomical scan within AFNI.

***fMRI data analysis.*** Analysis was then performed on regression coefficients from individual subject analyses using a 2 (Referential target: self or other) × 2 (Valence: positive or negative) × 2 (Intensity: low or high) repeated measures ANOVA. All regions were corrected for multiple comparisons via ClustSim (initial threshold: *p* < 0.001 corrected at *p* < 0.05 using an extent threshold of 12 voxels). Group effects were masked using a brain mask based on the mean normalized anatomical images of all participants.

After observing hypothesized effects, *post-hoc* analyses were performed to facilitate interpretations. For these analyses, average percent signal change was measured across all voxels within each ROI generated from the functional mask, and data were analyzed using appropriate follow-up tests within SPSS.

***Behavioral analysis.*** The evaluations made by the participants were re-coded into numerical values (completely disagree = 1, disagree = 2, agree = 3, completely agree = 4). Three 2 (Referential target: self or other) × 2 (Valence: positive or negative) × 2 (Intensity: low or high) repeated measures ANOVAs were conducted on the participant's judgment (their agreement with the statement), their RT and response consistency (i.e., response variance for each of the 8 stimulus classes), respectively.

### Study 2: personality traits questionnaire

**Figure A1:**
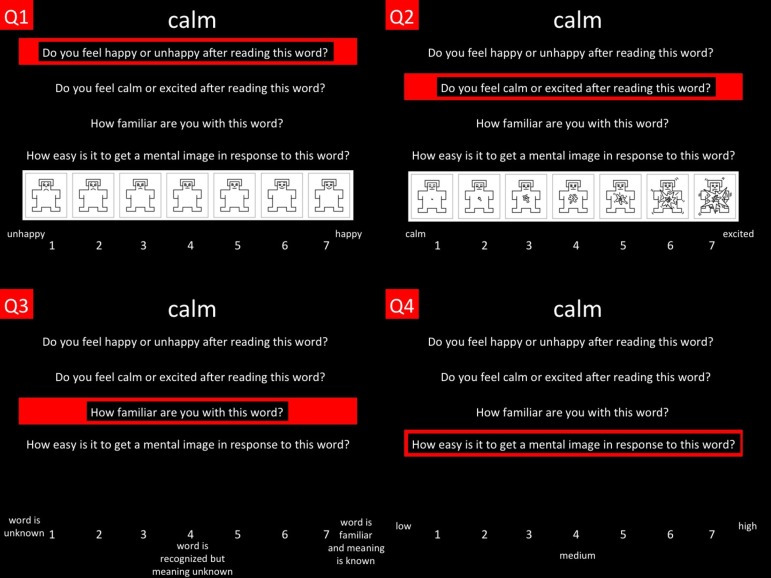
**Word rating questionnaire Study 2.** Screen shots of the four questions (Q1, Q2, Q3, and Q4) that were asked for each personality trait.

A group of 12 participants rated valence, arousal, familiarity, and imageability for every personality trait on a 7-point scale, using a computerized questionnaire (Figure A1). Personality traits were presented as words in random order. Each word was presented until the participant had provided a rating response for that word. Before filling out the questionnaire, participants read a manual that provided explanations on item scoring (Text A1).

Four 2 (Intensity: high or low) × 2 (Valence: positive or negative) ANOVAs were conducted on the valence, arousal, familiarity, and intensity ratings. In addition, we calculated the correlation between the valence ratings and the original likeableness scores of Anderson (Anderson, 1968).

## Results

### Study 1: referential processing

#### Behavioral data (see Table 1)

A 2 (Referential target: self or other) × 2 (Valence: positive or negative) × 2 (Intensity: low or high) repeated measures ANOVA was conducted on the participants' judgments. There was a main effect of Valence [*F*_(1, 20)_ = 331.94, *p* < 0.001]. Participants agreed more strongly with positive than negative traits. Additional to this was a Valence-by-Intensity interaction [*F*_(1, 20)_ = 184.48, *p* < 0.001]. Participants agreed more with high intensity than low intensity positive statements (*M*[High-Pos] = 2.86, *M*[Low-Pos] = 2.52; *t*_(20)_ = 14.13, *p* < 0.001) but with low intensity negative statements more than high intensity negative statements (*M*[High-Neg] = 1.23, *M*[Low-Neg] = 1.73; *t*_(20)_ = 8.76, *p* < 0.001). There were no effects of Referential target, or Intensity and no interaction effects of Referential target-by-Valence, Referential target-by-Intensity or Referential target-by-Intensity-by-Valence.

**Table 1:** **Behavioral data: Means and standard deviations (in brackets) for judgment (i.e., the level of agreement with the statement ranging from 1[completely disagree] to 4[completely agree]), RT, response variability (i.e., the variance in judgments), and the number of missed trials by condition**.

		**Self**		**Other**	
		**Low (±SD)**	**High (±SD)**	**Low (±SD)**	**High (±SD)**
Judgment	Positive	2.76 (±0.15)	3.15 (±0.21)	2.71 (±0.16)	3.19 (±0.30)
	Negative	2.14 (±0.27)	1.75 (±0.21)	2.04 (±0.19)	1.73 (±0.29)
RT (ms)	Positive	1827 (±248)	1741 (±261)	2044 (±283)	1910 (±290)
	Negative	1914 (±261)	1791 (±223)	2118 (±248)	1936 (±287)
Response variability	Positive	0.50 (±0.25)	0.20 (±0.14)	0.51 (±0.31)	0.22 (±0.09)
	Negative	0.50 (±0.33)	0.37 (±0.19)	0.33 (±0.24)	0.20 (±0.11)
No. Misses	Positive	0.67 (±1.20)	0.48 (±0.98)	1.05 (±1.28)	0.81 (±0.98)
	Negative	1.10 (±1.30)	1.00 (±1.18)	1.48 (±1.78)	1.00 (±1.18)

The ANOVA for participant RTs identified main effects of Referential target, Intensity, and Valence [*F*_(1, 20)_ = 23.04, 113.62, 6.26; *p* < 0.001, 0.001 and <0.05, respectively]. Participants were faster when making judgments about the self relative to the other (current US President), high intensity items relative to low intensity items and positive traits relative to negative traits. There were no significant interactions between any of the variables.

The ANOVA for response variability (i.e., the variability in response as a function of stimulus type) revealed a main effect of Intensity [*F*_(1, 20)_ = 29.93, *p* < 0.001]. Participants showed greater response variability in their judgments of low relative to high intensity items. In addition, there was a significant Valence-by-Intensity interaction [*F*_(1, 20)_ = 23.56, *p* < 0.001]. Response variability was higher for negative relative to positive high intensity judgments [*t*_(20)_ = 5.24, *p* < 0.001] but response variability for negative low intensity judgments did not differ from positive low intensity judgments[*t*_(20)_ = 1.96, *p* = 0.064]. Finally, there was also a significant Referential target-by-Valence interaction [*F*_(1, 20)_ = 36.27, *p* < 0.001]. Response variability was higher for self negative judgment trials relative to other negative trials [*t*_(20)_ = 3.31, *p* < 0.003], but there was no difference in response variability for self positive relative to other positive trials [*t*_(20)_ = 0.33, *p* = 0.743].

#### MRI results

The ANOVA conducted on the BOLD response data revealed regions showing significant main effects of Referential target, Valence, and Intensity as well as regions showing an interaction of Valence-by-Intensity.

**Figure 1:**
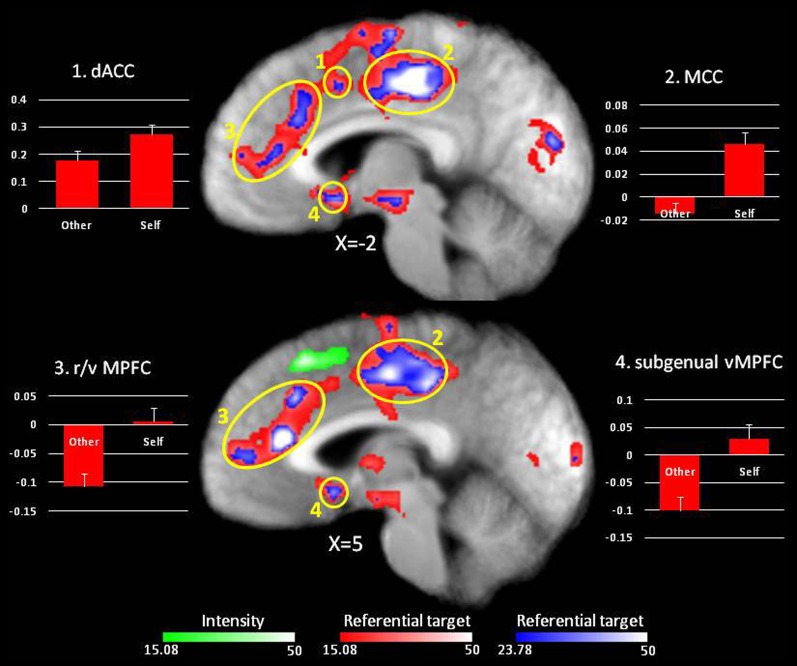
**Main effect of Referential target.** In red, regions showing an effect of Referential target at *p* < 0.001 (*F* = 15.08). Regions in green display an effect of Intensity at *p* < 0.001 (*F* = 15.08). Graphs depict percent signal change compared to fixation for local maxima using a height threshold of *p* < 0.0001 (*F* = 23.78, local maxima for Referential target are overlaid in blue).

***Main effect of Referential target.*** The main effect of Referential target identified an extended region that included local maxima using a height threshold of *p* < 0.0001 (*F* = 23.78) within vMPFC, dMPFC, rostral and dorsal ACC and ventral and dorsolateral prefrontal cortex (dlPFC) (see Table 2 and Figure 1, regions in red). All these local maxima displayed a larger BOLD response when making judgments about the self relative to the other. Notably though, only the dorsal part of the MPFC was significantly activated for both self-referential and other-referential processing, whereas more ventral regions (ACC and subgenual vMPFC) were deactivated during other-referential processing and did not show significant activation during self-referential processing (see Table 2 and Figure 1).

**Table 2:** **Main effect of Referential target**.

**Region**	***BA***	**L/R**	Coordinates of peak activation			***F***	**Voxels**	***Post-hoc* tests**		
			***x***	***y***	***z***			**Self**	**Other**	**Self vs. Other**
**CENTRAL MIDLINE STRUCTURES**										
Middle cingulate cortex	31	Right	4.5	−22.5	44.5	116.8	5985	>Fix	–	Self > Other
Dorsal anterior cingulate	32	Right	4.5	7.5	38.5	34.8		>Fix	>Fix	Self > Other
r/v MPFC	24	Left	−1.5	31.5	14.5	76.9		–	<Fix	Self > Other
Subgenual vMPFC	25	Right	4.5	10.5	−6.5	43.5		–	<Fix	Self > Other
Precuneus	7	Right	13.5	−55.5	50.5	23.8	24	>Fix		Self > Other
**LATERAL PREFRONTAL**										
dlPFC	10	Right	22.5	43.5	23.5	62.3		–	<Fix	Self > Other
dlPFC	6	Right	19.5	4.5	47.5	33.9		–	<Fix	Self > Other
dlPFC	9	Right	37.5	16.5	35.5	66.9		>Fix	–	
dlPFC	8	Left	−22.5	19.5	44.5	44.9	136	>Fix	–	Self > Other
Inferior frontal gyrus	6	Right	52.5	−4.5	20.5	39.4		>Fix	–	Self > Other
Precentral gyrus	6	Right	46.5	−1.5	41.5	27.5	20	>Fix	>Fix	Self > Other
**VISUAL CORTEX**										
Lingual gyrus	18	Right	10.5	−67.5	−0.5	95.7	315	>Fix	>Fix	Other > Self
Lingual gyrus	17	Left	−4.5	−88.5	5.5	39.4	42	>Fix	>Fix	Self > Other
Lingual gyrus	18	Left	−4.5	−73.5	5.5	49.5	99	>Fix	>Fix	Other > Self
**CEREBELLUM**										
Cerebellum	–	Right	10.5	−52.5	−21.5	28.8	36	>Fix	>Fix	Self > Other
Cerebellum	–	Left	−28.5	−40.5	−27.5	39.8	45	>Fix	>Fix	Self > Other
Cerebellum	–	Left	−25.5	−61.5	−42.5	21.5	17	>Fix	–	Self > Other
Cerebellum	–	Right	16.5	−58.5	−45.5	20.6	16	>Fix	–	Self > Other
Inferior temporal gyrus	37	Right	49.5	−46.5	−12.5	22.4	26	>Fix	–	Self > Other
Posterior insula	13	Left	−37.5	−31.5	20.5	75.1		>Fix	–	Self > Other
Caudate	–	Right	16.5	13.5	8.5	35.7		>Fix	–	Self > Other
Brainstem	–	Right	4.5	−16.5	−6.5	38.5		>Fix	–	Self > Other
Rolandic operculum	44	Right	46.5	−1.5	5.5	66.9		>Fix	<Fix	Self > Other
Middle temporal gyrus	37	Right	52.5	−58.5	2.5	19.7	22	–	<Fix	Self > Other
Superior temporal gyrus	42	Right	55.5	−31.5	14.5	73.7		–	<Fix	Self > Other
Postcentral gyrus	¾	Right	10.5	−37.5	65.5	20.1	20	–	–	Self > Other
Substantia nigra	–	Right	16.5	−22.5	−15.5	25.2	13	–	–	Self > Other

Thresholded at p = 0.001 uncorrected and a cluster extent threshold of 12 voxels. Gray text indicates local maxima using a height threshold of p < 0.0001 for the largest cluster of 5985 voxels (F = 23.78). Results of post-hoc tests are reported for every region displaying Referential target effect (thresholded at p = 0.05 uncorrected).

**Figure 2:**
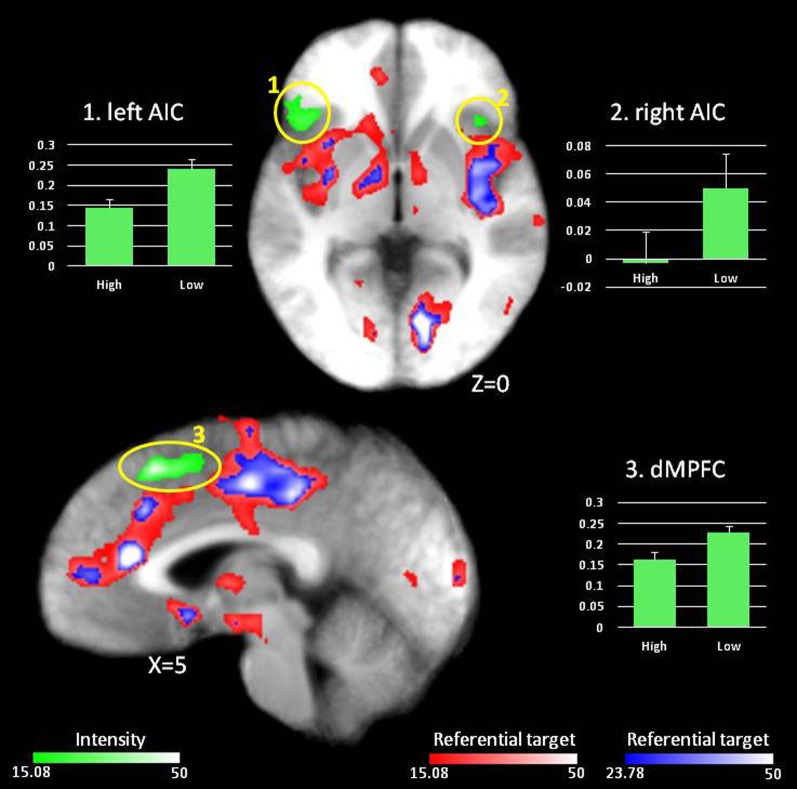
**Main effect of Intensity.** In red, regions showing an effect of Referential target at *p* < 0.001 (*F* = 15.08). Regions in green display an effect of Intensity at *p* < 0.001 (*F* = 15.08). Graphs depict percent signal change compared to fixation for regions displaying an effect of Intensity.

***Main effect of Intensity.*** A main effect of Intensity was observed in dMPFC as well as an extensive region of lateral frontal cortex (both inferior and superior) and bilateral anterior insula (AIC) (see Figure 2, regions in green). In all cases, BOLD responses were greater when making judgments about low relative to high intensity items (see Table 3 and Figure 2).

**Table 3:** **Brain regions displaying an effect of Intensity, Valence, and Valence-by-Intensity**.

**Region**	***BA***	**L/R**	**Coordinates of peak activation**			***F***	**Voxels**	***Post-hoc* tests**		
			***x***	***y***	***z***					
**Intensity**								**Low**	**High**	**Low vs. High**
dMPFC	8	Left	−4.5	19.5	47.5	52.4	119	>Fix	>Fix	Low > High
dlPFC	6	Left	−31.5	4.5	50.5	29.2	153	>Fix	>Fix	Low > High
AIC	45	Left	−49.5	31.5	5.5	49.9	119	>Fix	>Fix	Low > High
	47	Right	37.5	22.5	−0.5	21.4	12	–	–	Low > High
**Valence**								**Pos**	**Neg**	**Pos vs. Neg**
Posterior insula	13	Left	−34.5	−19.5	14.5	22.8	15	–	<Fix	Pos > Neg
	13	Right	46.5	−19.5	14.5	31.8	30	–	>Fix	Neg > Pos
Primary somato-sensory cortex	3	Left	−34.5	−28.5	50.5	152.8	683	>Fix	–	Pos > Neg
	3	Right	34.5	−25.5	50.5	84.5	664	–	>Fix	Neg > Pos
Cerebellum	–	Left	−16.5	−49.5	−18.5	44.3	80	>Fix	>Fix	Neg > Pos
	–	Right	16.5	−49.5	−12.5	55.7	150	>Fix	>Fix	Pos > Neg
Middle cingulate gyrus	6	Left	−10.5	−13.5	50.5	27.6	58	>Fix	<Fix	Pos > Neg
Thalamus	–	Left	−16.5	−22.5	5.5	37.1	23	>Fix	>Fix	Pos > Neg
**Valency-by-Intensity**										
Primary somato-sensory cortex	2	Right	40.5	−25.5	47.5	39.2	288			
	3	Left	−31.5	−25.5	44.5	33.8	97			

Thresholded at p = 0.001 uncorrected and a cluster extent threshold of 12 voxels. Results of post-hoc tests are reported for every region displaying an Intensity or Valence effect (thresholded at p = 0.05 uncorrected).

***Main effect of Valence.*** There was a main effect of valence within bilateral posterior insula, of which the left posterior insula was recruited more strongly for positive valenced traits and the right posterior insula for negative valences traits (see Table 3). A similar laterality effect was observed in bilateral primary and somatosensory cortex (SI).

***Interaction effect of Valence-by-Intensity.*** Bilateral SI displayed a valence-by-intensity effect (see Table 3); right SI was recruited for negatively valenced traits and for the negative traits most strongly for those with a high intensity, left SI was recruited for positive traits and also most strongly for those traits with a high intensity.

***Conjunction analysis.*** The degree of overlap was determined in the regions identified by the main effect of Referential target and those identified by the main effect of Intensity. Specifically, we wished to determine whether the regions showing increased activity when making judgments about the self might overlap with those implicated for more difficult judgments. This conjunction analysis revealed minimal overlap between the Referential target effect in dMPFC (6 out of 119 voxels). However, none of the other regions displaying an effect of Intensity (dlPFC and bilateral AIC) overlapped with regions displaying a Referential target effect.

### Study 2: personality traits questionnaire

Given concerns regarding the valence, arousal, familiarity, and imageability of the personality trait words, we asked 12 participants to provide these ratings for each of the words. We then conducted four independent 2 (Intensity: high or low) × 2 (Valence: positive or negative) ANOVAs on the valence, arousal, familiarity, and intensity ratings to determine whether the stimulus categories were associated with significant differences in these ratings. With respect to the valence ANOVA there was, unsurprisingly a highly significant effect [*F*_(1, 60)_ = 178.87, *p* < 0.001]. Positive words [as identified by Anderson's (1968) likeability ratings] were rated as more positive in valence than negative words (*M*[Pos] = 5.33, *M*[Neg] = 2.64; *t*_(62)_ = 11.803, *p* < 0.001). In addition, there was an unsurprising significant Valence-by-Intensity interaction [*F*_(1, 60)_ = 18.59, *p* < 0.001]; High negative words were rated as less positive than low negative words [*M* = 2.30 vs. 2.97; *t*_(30)_ = 2.50, *p* = 0.018] while high positive words were rated as more positive than low positive words [*M* = 5.87 vs. 4.80; *t*_(30)_ = 3.54, *p* = 0.001].

Due to concerns that there might be many possible relations between “likeableness” (ratings provided by Anderson (1968) and emotional valence (and arousal), we also conducted a correlation analysis between the 12 participant's valence ratings and the likeableness scores provided by Anderson (1968). This revealed a highly significant Pearson correlation of 0.929 (*p* < 0.001). However, emotional valence ratings did not correlate with arousal ratings (*r* = 0.059; n.s.).

With respect to arousal ratings, there was no main effect of either Valence or Intensity or significant interaction. There was, however, a main effect of Valence for familiarity ratings [*F*_(1, 60)_ = 4.52, *p* = 0.038]. Words in the positive category were rated as more familiar relative to words in the negative category (*M*[Pos] = 6.82, *M*[Neg] = 6.61; *t*_(62)_ = 2.07, *p* < 0.042). There was also a main effect of Intensity on the imageability ratings [*F*_(1, 60)_ = 4.27, *p* = 0.043]. High intensity words were rated as more imageable than low intensity words (*M*[High] = 6.09, *M*[Low] = 5.71; *t*_(62)_ = 2.08, *p* < 0.041).

## Discussion

The present study investigated the influence of valence and decision difficulty on regions implicated in self-referential processing. There were five main findings: First, ventral, rostral, and dorsal parts of MPFC showed greater activity when subjects judged traits about themselves relative to judging traits about the current US President. Second, none of these regions showed significant modulation by trait valence, though a significant main effect of Valence was seen within bilateral posterior insula. Third, although all regions in MPFC were relatively stronger recruited for self, only dorsal parts were significantly activated for both self-referential and other-referential processing. Fourth, with respect to decision difficulty, both the bilateral AIC and the dMPFC were more strongly recruited when participants were evaluating traits of low intensity. Fifth, with the exception of a very small region of dMPFC, the regions showing a main effect of Referential target did *not* overlap with those regions responsive to a parameter relating to task difficulty in trait judgments; item intensity.

Participants showed increased activity within ventral, rostral, and dorsal parts of MPFC, right IFG and dlPFC when making judgments about themselves relative to another individual (the current US President). These findings are consistent with a large body of previous work examining self-referential processing (van der Meer et al., 2010; Northoff et al., 2011). A ventral-dorsal division was observed in the MPFC: *Post-hoc* analyses indicated that dorsal regions were significantly recruited during both self- and other-referential processing (albeit more so during self-referential processing), whereas ventral parts of the MPFC did not show significant activations (over baseline) when either engaged by either self- or other-referential processing. Instead, vMPFC showed deactivation during other-referential processing. In short, the observed Referential target effect in dorsal regions was due to a stronger activation during self while the Referential target effect in ventral regions was due to a deactivation when making judgments about the other. This pattern of activations (at or below baseline during self-referential processing and even more below baseline during other-referential processing) has been observed in many studies employing similar paradigms (Kelley et al., 2002; Heatherton et al., 2006; D'Argembeau et al., 2007; Gutchess et al., 2007; Jenkins and Mitchell, 2011) though not by all (Kircher et al., 2000; D'Argembeau et al., 2005; Vanderwal et al., 2008; Moran et al., 2011; Sul et al., 2011). It has been argued that high vMPFC activity during rest potentially represents self-reflective processes that are reduced when representing the other (e.g., Kelley et al., 2002; D'Argembeau et al., 2005; Heatherton et al., 2006; Jenkins and Mitchell, 2011). Of course, this assumes that self-reflective processes are rapidly engaged in the absence of task relevant stimuli and that these are comparable (albeit non-conscious and non-task driven) to those initiated when asking the individual to engage in self-referential judgments.

Previous work has examined the impact of affect on self-referential processing of personality traits (Fossati et al., 2003; Moran et al., 2006; Gutchess et al., 2007). While there is notable overlap between regions engaged in self-referential processing and those associated to reward related processing during performance of a gambling task (de Greck et al., 2008; Enzi et al., 2009), there has been little evidence of significant modulation by valence when engaged in self-referential processing. One study reported an interaction between self-referential processing of personality traits and valence within a more ventral region of MPFC (Moran et al., 2006). However, that has not been seen subsequently (Fossati et al., 2003; Phan et al., 2004; Gutchess et al., 2007; Northoff et al., 2009) and nor was it seen here. On the basis of these results, we assume that emotional responding plays a relatively minor role in self-referential processing, even if affect information has an impact (as evidenced by the judgment, RT and response variability behavioral data). Given the absence of any emotional response at the neural level, we speculate that this affect information is represented in semantic systems that are engaged when making self- and other-referential judgments.

But what are the processes engaged by self/other-referential processing tasks? It could be argued that such tasks are variants of standard sentence verification tasks (e.g., “A canary is a bird”) where self-referential trials involve the subject presumably “knowing” while in other-referential trials they can only “guess” it (anonymous reviewer's suggestion). Certainly, sentence verification tasks elicit activity within dorsomedial and lateral frontal cortices (Sanjuan et al., 2010). However, the activity of these regions is greater for sentences which compare words with less shared features than for those comparing words with more shared features (Raposo et al., 2012). Moreover, findings for the conceptually similar context verification task reported that medial and lateral frontal cortices showed greater activity during ambiguous relative to unambiguous trials (Hoenig and Scheef, 2009). Since self-referential statements would appear to have more shared features and are less ambiguous than other-referential judgments, one might have expected them to be associated with associated with less activity within dmPFC than other-referential statements on the basis of this literature. Given that in this study they are not, the hypothesis that self-referential processing tasks are variants of standard sentence verification tasks where self-referential trials involve the subject presumably “knowing” the answer while in other referential trials they can only “guess” the answer, is not supported.

In the current study, decision difficulty was manipulated via trait intensity. In pilot work, participants found judgments concerning high intensity to be significantly easier than judgments concerning low intensity traits. Moreover, as was seen here, RT and variance in given responses was lower for high intensity traits relative to low intensity traits. Recent modeling work of the role of dmPFC in decision-making has suggested that this region maps the various response-option combinations elicited by a stimulus such that the greater the number of options, the greater the dmPFC will be seen (Alexander and Brown, 2011). This is consistent with previous findings in the decision making literature (e.g., Marsh et al., 2007). As such this model predicted that low intensity traits would be associated with greater dMPFC activity than high intensity traits, a prediction confirmed by the current results. It is also noteworthy that regions of lateral frontal and AIC were also more responsive to low relative to high intensity traits. This is consistent with suggestions that the response of dMPFC includes the organization of attentional resources via lateral frontal cortex and behavioral change via inferior frontal cortex/anterior insula cortex (Miller and Cohen, 2001; Budhani et al., 2007).

If this form of decision-making process, response outcome tracking, was related to self-referential processing then one would expect that the impact of Referential target (self vs. other) and decision difficulty (low intensity vs. high intensity) should show considerable overlap. However, this was only minimally seen (5% of the region identified by the main effect of decision difficulty was also shown to be responsive to referential target). Instead, our alternative hypothesis was supported; i.e., that largely separable regions of dMPFC are implicated in self-referential as opposed to decision difficulty (even decision difficulty concerning self-referential traits).

It could be argued that if the PRO models account of dmPFC was an account of this region's role in self-referential processing, then there should be greater activity within dmPFC for other-referential rather than self-referential judgments. This would be because participants presumably “know” (and thus have less response options) the degree to which it corresponds to the self but can only guess with respect to the other individual (anonymous reviewer's suggestion). However, it should be noted that there was no significant main effect of referential target with respect to response variability (see also Table 1). As such the PRO model should not necessarily predict differential dmPFC activity as a function of referential target. Of course, given this it's not clear that the model should predict greater activity for self relative to other either. As such, PRO cannot be considered a complete model of dmPFC activity (a large region of prefrontal cortex). However, it is a useful model of *one* of the functions of this region. Self-referential processing is another of these functions.

Recently a distinction has been drawn between internally and externally guided decision making (Nakao et al., 2012). It is argued that “instances of decision-making in which no correct answer based on external circumstances is available for the subject (internally guided decision-making) … are usually made … where the answer depends on the subject's own, i.e., internal, preferences rather than on external, i.e., circumstantial, criteria.” (Nakao et al., 2012, p. 1). Similar distinctions have been made before (e.g., Lieberman and Eisenberger, 2005; Volz et al., 2006). These reviews suggest that internally guided decision-making relies on MPFC (Lieberman and Eisenberger, 2005; Volz et al., 2006; Nakao et al., 2012), PCC and temporal cortex (Lieberman and Eisenberger, 2005; Nakao et al., 2012). A similar neural network showed greater activity during self-referential processing relative to other-referential processing in the current study. This could be taken to suggest that self-referential processing relies more on processes implicated in internally generated decision-making than other-referential processing; it is possible that one utilizes ones preferences more for self and external, circumstantial criteria more for other. However, as with all reverse inferences, this suggestion is open to challenge without direct empirical manipulation.

Two caveats should be considered with respect to the current results. First, study 2 indicated that our positive words were more familiar to the participants than the negative words. However, our relative absence of valence findings, beyond somatosensory regions, suggests that this confound had a limited impact on the BOLD response. Second, study 2 also indicated that our high intensity items had a higher imageability score than our low intensity items. Importantly, however, previous studies of the neural correlates of imageability (Bedny and Thompson-Schill, 2006) or contrasting concrete vs. abstract words (Jessen et al., 2000; Binder et al., 2005) have consistently implicated more lateral regions of superior frontal cortex than those implicated in the task difficulty intensity effect seen here. In addition, they have consistently implicated regions of parietal cortex and precuneus—neither of which was effected by intensity level in the current study. In short, it appears unlikely that our Intensity effects can be attributed to imageability differences of the word categories. There were no significant item effects on arousal and thus differences in this variable would not appear to be usefully explanatory regarding the current data.

Considerable work has been conducted on self-referential processing implicating a consistent network of brain regions including CMS, as well as inferior frontal cortex, insula and the temporal poles. However, much is still unknown about the different computations underlying self-referential processing and how they are implemented. In this study, we have examined the impact of two variables on regions implicated in self-referential processing: valence and decision difficulty. It will be important to examine the impact of other manipulations on these systems also.

In summary, the current study implicates a network of CMS in self-referential processing. Some regions, in particular dACC, are implicated in self- and other-referential processing (albeit showing stronger responses when self- relative to other-processing). Potentially, this represents executive organization of semantic representations, such that concepts can be examined with respect to the self or the other. Other regions, in particular vMPFC and subgenual ACC, may also be implicated in both self- and other-referential processing. However they are associated with significant deactivation during other-referential processing and non-significant activation during self-referential processing. While this may reflect self-referential processing during rest (e.g., Kelley et al., 2002; D'Argembeau et al., 2005; Heatherton et al., 2006; Jenkins and Mitchell, 2011), this assumes that self-reflective processes are rapidly engaged in the absence of task relevant stimuli (given the event-related design) and that they are comparable (albeit non-conscious and non-task driven) to those initiated when asking the individual to engage in self-referential judgments. Importantly, while there have been challenges that dMPFC activity during self-referential processing may reflect general inferential/decision making processes rather than self-referential processing *per se* (Legrand and Ruby, 2009), the current data suggest that regions of dMPFC implicated in trait decision difficulty (i.e., more general decision making processing) are independent of those implicated in self-referential processing.

### Conflict of interest statement

The authors declare that the research was conducted in the absence of any commercial or financial relationships that could be construed as a potential conflict of interest.

## Appendix

### Text A1: word rating questionnaire

#### General instructions

Thank you for participating in this study. We would like to know how you respond to certain words. For each word we will ask you four questions. Please rate each question on a scale of 1–7. When you press a number, the program will immediately jump to the next question. When making your ratings, try to be as accurate as possible, but do not spend too much time on any one word. We will now go over each question with two example words and explain how you should rate the questions. You can always refer to this information in the paper copy next to you.

#### Instructions question 1

Underneath the question you will see a set of figures called SAM, and you will be using these figures to rate how you felt while reading each word. For this question, SAM shows the feelings Unhappy vs. Happy, which ranges from a frown to a smile. At one extreme of this scale, you are happy, pleased, satisfied, contented, hopeful. When you feel completely happy you should indicate this by selecting the figure at the right. The other end of the scale is when you feel completely unhappy, annoyed, unsatisfied, melancholic, despaired, or bored. You can indicate feeling completely unhappy by selecting the figure on the left. The figures also allow you to describe intermediate feelings of pleasure, by selecting any of the other pictures. If you feel completely neutral, neither happy nor sad, select the figure in the middle. There are a total of 7 possible points along each rating scale that you can select to indicate the extent to which you felt happy or unhappy. You select the picture by pressing the number that is written underneath the picture (adapted from Bradley and Lang, 1999).

#### Instructions question 2

For this question, SAM shows the feelings Calm vs. Exited. At one extreme of this scale you are stimulated, excited, frenzied, jittery, wide-awake, or aroused. When you feel completely *aroused*, select the figure at the left of the row. Now look at the other end of the excited-calm scale, which is the completely opposite feeling. Here you would feel completely relaxed, calm, sluggish, dull, sleepy, or unaroused. Indicate feeling *calm* by selecting the figure at the right of the row. As with the happy–unhappy scale, you can represent intermediate levels of excitedness or calmness by selecting any of the other figures. If you are not excited nor at all calm, select the figure in the middle of the row (adapted from Bradley and Lang, 1999).

#### Instructions question 3

For this question we ask you to rate how familiar you are with this word. You can rate the familiarity on a 7-point scale, where a 1 indicates that the word is unknown to you. A rating of 7 indicates that the word is familiar to you and its meaning well known. A rating of 4 indicates that you definitely recognize the word, but do not know its meaning. The ratings 2, 3, 5, and 6 are used to indicate variations between these response categories. For example, a rating of 3 indicates that you might have seen the word before, while a rating of 5 indicates that you recognize the word but have only the vaguest notion of its meaning. You can indicate the word familiarity by pressing the appropriate number on the keyboard (adapted from Nusbaum et al., 1984).

#### Instructions question 4

Words differ in their capacity to arouse mental images of things or events. Some words arouse a sensory experience, such as a mental picture or sound, very quickly and easily whereas other words may do so only with difficulty (i.e., after a long delay) or not at all.

The purpose of this question is to rate the ease or difficulty with which this word arouses mental images. Any word, that in your estimation arouses a mental image (i.e., a mental picture or sound, or other sensory experience) very quickly and easily should be given a high imagery rating (at the upper end of the numerical scale). Any word that arouses a mental image with difficulty or not at all should be given a low imagery rating (at the lower end of the numerical scale). For example think of the word “BUSY.” “BUSY” would probably arouse an image (e.g., of someone running around all the time) relatively easily and would be rated as high. “INDIVIDUALISTIC” would probably do so with difficulty and be rated as low imagery.

Because words tend to make you think of other words as associates, it is important that your ratings not be based on this, and that you judge only the ease with which you get a mental image of an object or event in response to each word.

Your imagery ratings will be made on a 1–7 scale. A value of 1 will indicate a low imagery rating, and a value of 7 will indicate a high imagery rating. Values of 2–6 will indicate inter- mediate ratings (adapted from Cortese and Fugett, 2004).

**Table A1:** **Words used in paradigm**.

	**Condition**	**Likeableness**	**Meaningfullness**	**Valence**	**Arousal**	**Familiarity**	**Imageability**
Thoughtful	Pos-High	529	376	6	2.83	6.92	5.75
Kind	Pos-High	520	368	6.42	2.67	7	6.08
Good-humored	Pos-High	507	366	2.42	4.75	6.92	6.92
Well-spoken	Pos-High	501	332	5.75	3.67	6.75	6
Quick-witted	Pos-High	494	356	6	4.75	6.58	5.42
Brilliant	Pos-High	490	366	5	3.75	6.67	4.58
Bright	Pos-High	483	362	6.67	5.42	6.92	6.25
Alert	Pos-High	480	370	5	3.25	6.25	4.75
Spirited	Pos-High	477	342	5.75	4.33	6.75	5.83
Capable	Pos-High	471	370	2.17	3.58	6.92	6.42
Accurate	Pos-High	464	336	5.25	2.58	6.75	5.58
Generous	Pos-High	459	370	6.58	4.08	6.92	6.5
Inventive	Pos-High	453	356	6	4.58	6.75	6.08
Attentive	Pos-High	450	372	2.42	4.92	6.67	6.67
Amiable	Pos-High	446	348	5.5	3.75	6.92	6.17
Composed	Pos-High	439	340	2.83	3.5	6.42	5.58
Agreeable	Pos-Low	434	354	4.83	4.92	6.83	6.42
Decisive	Pos-Low	427	360	4.42	3.83	6.75	4.83
Light-hearted	Pos-Low	424	324	5.67	3.08	6.92	5.67
Outgoing	Pos-Low	412	364	6.33	5.08	6.92	6.67
Hopeful	Pos-Low	406	328	6.25	4	6.92	5.17
Orderly	Pos-Low	399	360	5.5	3.58	7	6.58
Candid	Pos-Low	389	316	6.17	3.83	6.83	5.83
Soft-spoken	Pos-Low	380	354	4.08	2.42	6.83	6.58
Sentimental	Pos-Low	371	360	5.42	2.75	6.83	6.17
Subtle	Pos-Low	365	320	4.17	2.75	6.83	4.75
Talkative	Pos-Low	352	390	5.5	4.42	6.92	6.83
Deliberate	Pos-Low	345	344	3.25	5	6.83	6.5
Forward	Pos-Low	318	346	6.08	4.25	6.5	6.58
Conservative	Pos-Low	295	352	2.25	4	6.92	6.42
Unlucky	Pos-Low	280	360	2.25	3.75	7	5.92
Naive	Pos-Low	270	360	3.5	3.25	6.75	5.92
Extravagant	Neg-Low	263	360	2	4.83	7	6.67
Daydreamer	Neg-Low	260	368	5.25	3.75	6.83	5.25
Self-conscious	Neg-Low	249	366	3.08	3.58	6.92	6
Conformist	Neg-Low	241	372	3.5	3.42	6.92	6.5
Overcautious	Neg-Low	229	360	2.67	3.5	6.75	5.42
Forgetful	Neg-Low	224	386	4.67	3.67	6.75	5.83
Spendthrift	Neg-Low	221	354	3.5	3.08	5.08	4.08
Fearful	Neg-Low	214	370	3	3.67	6.92	5.5
Sad	Neg-Low	209	358	1.33	2.83	7	7
Demanding	Neg-Low	203	362	5.17	5.25	6.67	6.92
Obstinate	Neg-Low	197	348	3.33	3.25	4.58	3.58
Unobservant	Neg-Low	194	366	3	3.17	6.33	5
Illogical	Neg-Low	186	354	2.5	3.83	6.58	4.75
Unwise	Neg-Low	180	358	2.42	3	6.83	5.17
Listless	Neg-Low	169	332	3	3.5	5.5	4.92
Pessimistic	Neg-Low	164	376	1.58	4.08	6.92	6.17
Smug	Neg-High	161	304	2.83	3.67	6.33	5.25
Hot-headed	Neg-High	158	362	2.25	4.83	6.75	6.5
Sloppy	Neg-High	153	376	2.92	3.75	6.92	6.83
Unruly	Neg-High	150	324	2.58	4.33	6.5	5.75
Uninteresting	Neg-High	146	372	2.58	2.42	6.92	6.08
Careless	Neg-High	140	374	5.33	2.67	6.92	6.42
Irrational	Neg-High	130	354	2.67	4.5	6.83	5.58
Gossipy	Neg-High	119	376	5.67	3.58	7	5.17
Cowardly	Neg-High	110	374	4.83	2.67	6.92	6.67
Irresponsible	Neg-High	106	372	2.25	4.08	6.83	6.25
Humorless	Neg-High	101	362	2.08	2.83	6.58	5.67
Ill-mannered	Neg-High	95	374	2.25	4	6.83	5.83
Belligerent	Neg-High	86	332	6.17	4.33	6.92	6.67
Heartless	Neg-High	78	350	1.67	3.58	6.92	6.5
Insincere	Neg-High	66	364	1.75	3.67	6.58	5.5
Mean	Neg-High	37	356	1.75	4.42	6.92	6.67

Likeableness and meaningfulness scores as reported by Anderson (1968). Condition as used in the current paradigm. Valence, arousal, familiarity, and imageability scores as reported by an independent group of 12 participants.

## References

[B1] Alexander W. H. Brown J. W. (2011). Medial prefrontal cortex as an action-outcome predictor. Nat. Neurosci. 14, 1338–1344 10.1038/nn.2921 21926982 PMC3183374

[B2] American Psychiatric Association. (1994). Diagnostic and Statistical Manual of Mental Disorders (Dsm-Iv). Washington, DC: American Psychiatric Association

[B3] Amodio D. M. Frith C. D. (2006). Meeting of minds: the medial frontal cortex and social cognition. Nat. Rev. Neurosci. 7, 268–277 10.1038/nrn1884 16552413

[B4] Anderson N. H. (1968). Likableness ratings of 555 personality-trait words. J. Pers. Soc. Psychol. 9, 272–279 5666976 10.1037/h0025907

[B5] Bedny M. Thompson-Schill S. L. (2006). Neuroanatomically separable effects of imageability and grammatical class during single-word comprehension. Brain Lang. 98, 127–139 10.1016/j.bandl.2006.04.008 16716387

[B6] Binder J. R. Westbury C. F. McKiernan K. A. Possing E. T. Medler D. A. (2005). Distinct brain systems for processing concrete and abstract concepts. J. Cogn. Neurosci. 17, 905–917 16021798 10.1162/0898929054021102

[B7] Botvinick M. M. Cohen J. D. Carter C. S. (2004). Conflict monitoring and anterior cingulate cortex: an update. Trends Cogn. Sci. 8, 539–546 10.1016/j.tics.2004.10.003 15556023

[B8] Budhani S. Marsh A. A. Pine D. S. Blair R. J. (2007). Neural correlates of response reversal: considering acquisition. Neuroimage 34, 1754–1765 10.1016/j.neuroimage.2006.08.060 17188518

[B9] Cox R. W. (1996). Afni: software for analysis and visualization of functional magnetic resonance neuroimages. Comput. Biomed. Res. 29, 162–173 8812068 10.1006/cbmr.1996.0014

[B10] Craik F. I. M. Moroz T. M. Moscovitch M. Stuss D. T. Winocur G. Tulving E. (1999). In search of the self: a positron emission tomography study. Psychol. Sci. 10, 26–34

[B11] D'Argembeau A. Collette F. Van der Linden M. Laureys S. Del Fiore G. Degueldre C. (2005). Self-referential reflective activity and its relationship with rest: a pet study. Neuroimage 25, 616–624 10.1016/j.neuroimage.2004.11.048 15784441

[B12] D'Argembeau A. Ruby P. Collette F. Degueldre C. Balteau E. Luxen A. (2007). Distinct regions of the medial prefrontal cortex are associated with self-referential processing and perspective taking. J. Cogn. Neurosci. 19, 935–944 10.1162/jocn.2007.19.6.935 17536964

[B13] de Greck M. Rotte M. Paus R. Moritz D. Thiemann R. Proesch U. (2008). Is our self based on reward? self-relatedness recruits neural activity in the reward system. Neuroimage 39, 2066–2075 10.1016/j.neuroimage.2007.11.006 18155927

[B14] Enzi B. de Greck M. Prosch U. Tempelmann C. Northoff G. (2009). Is our self nothing but reward? Neuronal overlap and distinction between reward and personal relevance and its relation to human personality. PLoS ONE 4:e8429 10.1371/journal.pone.0008429 20041155 PMC2794541

[B15] Etkin A. Egner T. Kalisch R. (2011). Emotional processing in anterior cingulate and medial prefrontal cortex. Trends Cogn. Sci. 15, 85–93 10.1016/j.tics.2010.11.004 21167765 PMC3035157

[B16] Fellows L. K. Farah M. J. (2005). Is anterior cingulate cortex necessary for cognitive control? Brain 128(Pt 4), 788–796 10.1093/brain/awh405 15705613

[B17] First M. B. Gibbon R. L. Williams J. B. W. Benjamin L. S. (1997). Structured Clinical Interview for Dsm-Iv Axis Ii Personality Disorders (Scid-Ii). Washington, DC: American Psychiatric Press, Inc

[B18] Fossati P. Hevenor S. J. Graham S. J. Grady C. Keightley M. L. Craik F. (2003). In search of the emotional self: an fmri study using positive and negative emotional words. Am. J. Psychiatry 160, 1938–1945 10.1176/appi.ajp.160.11.1938 14594739

[B19] Grinband J. Savitskaya J. Wager T. D. Teichert T. Ferrera V. P. Hirsch J. (2011). The dorsal medial frontal cortex is sensitive to time on task, not response conflict or error likelihood. Neuroimage 57, 303–311 10.1016/j.neuroimage.2010.12.027 21168515 PMC3114292

[B20] Gutchess A. H. Kensinger E. A. Schacter D. L. (2007). Aging, self-referencing, and medial prefrontal cortex. Soc. Neurosci. 2, 117–133 10.1080/17470910701399029 18633811

[B21] Heatherton T. F. Wyland C. L. Macrae C. N. Demos K. E. Denny B. T. Kelley W. M. (2006). Medial prefrontal activity differentiates self from close others. Soc. Cogn. Affect. Neurosci. 1, 18–25 10.1093/scan/nsl001 18985097 PMC2555408

[B22] Hoenig K. Scheef L. (2009). Neural correlates of semantic ambiguity processing during context verification. Neuroimage 45, 1009–1019 10.1016/j.neuroimage.2008.12.044 19167505

[B23] Jasinska A. J. Yasuda M. Rhodes R. E. Wang C. Polk T. A. (2012). Task difficulty modulates the impact of emotional stimuli on neural response in cognitive-control regions. Front. Psychol. 3:345 10.3389/fpsyg.2012.00345 23060828 PMC3464044

[B24] Jenkins A. C. Mitchell J. P. (2011). Medial prefrontal cortex subserves diverse forms of self-reflection. Soc. Neurosci. 6, 211–218 10.1080/17470919.2010.507948 20711940

[B25] Jessen F. Heun R. Erb M. Granath D. O. Klose U. Papassotiropoulos A. (2000). The concreteness effect: evidence for dual coding and context availability. Brain Lang. 74, 103–112 10.1006/brln.2000.2340 10924219

[B26] Kalbfleisch M. L. Van Meter J. W. Zeffiro T. A. (2007). The influences of task difficulty and response correctness on neural systems supporting fluid reasoning. Cogn. Neurodyn. 1, 71–84 10.1007/s11571-006-9007-4 19003497 PMC2288952

[B27] Kelley W. M. Macrae C. N. Wyland C. L. Caglar S. Inati S. Heatherton T. F. (2002). Finding the self? an event-related fmri study. J. Cogn. Neurosci. 14, 785–794 10.1162/08989290260138672 12167262

[B28] Kircher T. T. J. Senior C. Phillips M. L. Benson P. J. Bullmore E. T. Brammer M. (2000). Towards a functional neuroanatomy of self processing: effects of faces and words. Brain Res. Cogn. Brain Res. 10, 133–144 10.1016/S0926-6410(00)00036-7 10978701

[B29] Legrand D. Ruby P. (2009). What is self-specific? Theoretical investigation and critical review of neuroimaging results. Psychol. Rev. 116, 252–282 10.1037/a0014172 19159156

[B30] Lieberman M. D. Eisenberger N. I. (2005). Conflict and habit: a social cognitive neuroscience approach to the self, in On Building, Defending and Regulating the Self: A Psychological Perspective, eds Tesser A. Wood J. V. Stapel D. A. (New York, NY: Psychology Press), 77–102

[B31] Lindquist K. A. Wager T. D. Kober H. Bliss-Moreau E. Barrett L. F. (2012). The brain basis of emotion: a meta-analytic review. Behav. Brain Sci. 35, 121–143 10.1017/S0140525X11000446 22617651 PMC4329228

[B32] Livesey A. C. Wall M. B. Smith A. T. (2007). Time perception: manipulation of task difficulty dissociates clock functions from other cognitive demands. Neuropsychologia 45, 321–331 10.1016/j.neuropsychologia.2006.06.033 16934301

[B33] Lythe K. E. Williams S. C. Anderson C. Libri V. Mehta M. A. (2012). Frontal and parietal activity after sleep deprivation is dependent on task difficulty and can be predicted by the fmri response after normal sleep. Behav. Brain Res. 233, 62–70 10.1016/j.bbr.2012.04.050 22565029

[B34] Marsh A. A. Blair K. S. Vythilingam M. Busis S. Blair R. J. (2007). Response options and expectations of reward in decision-making: the differential roles of dorsal and rostral anterior cingulate cortex. Neuroimage 35, 979–988 10.1016/j.neuroimage.2006.11.044 17292631 PMC1868682

[B35] Miller E. K. Cohen J. D. (2001). An integrative theory of prefrontal cortex function. Annu. Rev. Neurosci. 24, 167–202 10.1146/annurev.neuro.24.1.167 11283309

[B36] Mitchell J. P. Macrae C. N. Banaji M. R. (2006). Dissociable medial prefrontal contributions to judgments of similar and dissimilar others. Neuron 50, 655–663 10.1016/j.neuron.2006.03.040 16701214

[B37] Moran J. M. Lee S. M. Gabrieli J. D. (2011). Dissociable neural systems supporting knowledge about human character and appearance in ourselves and others. J. Cogn. Neurosci. 23, 2222–2230 10.1162/jocn.2010.21580 20946059

[B38] Moran J. M. Macrae C. N. Heatherton T. F. Wyland C. L. Kelley W. M. (2006). Neuroanatomical evidence for distinct cognitive and affective components of self. J. Cogn. Neurosci. 18, 1586–1594 10.1162/jocn.2006.18.9.1586 16989558

[B39] Nakao T. Ohira H. Northoff G. (2012). Distinction between externally vs. internally guided decision-making: operational differences, meta-analytical comparisons and their theoretical implications. Front. Neurosci. 6:31 10.3389/fnins.2012.00031 22403525 PMC3293150

[B40] Northoff G. Qin P. Feinberg T. E. (2011). Brain imaging of the self-conceptual, anatomical and methodological issues. Conscious. Cogn. 20, 52–63 10.1016/j.concog.2010.09.011 20932778

[B41] Northoff G. Schneider F. Rotte M. Matthiae C. Tempelmann C. Wiebking C. (2009). Differential parametric modulation of self-relatedness and emotions in different brain regions. Hum. Brain Mapp. 30, 369–382 10.1002/hbm.20510 18064583 PMC6870760

[B42] Paus T. Koski L. Caramanos Z. Westbury C. (1998). Regional differences in the effects of task difficulty and motor output on blood flow response in the human anterior cingulate cortex: a review of 107 pet activation studies. Neuroreport 9, R37–R47 9674567 10.1097/00001756-199806220-00001

[B43] Phan K. L. Taylor S. F. Welsh R. C. Ho S. H. Britton J. C. Liberzon I. (2004). Neural correlates of individual ratings of emotional salience: a trial-related fmri study. Neuroimage 21, 768–780 10.1016/j.neuroimage.2003.09.072 14980580

[B44] Phan K. L. Wager T. D. Taylor S. F. Liberzon I. (2004). Functional neuroimaging studies of human emotions. CNS Spectr. 9, 258–266 15048050 10.1017/s1092852900009196

[B45] Powell L. J. Macrae C. N. Cloutier J. Metcalfe J. Mitchell J. P. (2010). Dissociable neural substrates for agentic versus conceptual representations of self. J. Cogn. Neurosci. 22, 2186–2197 10.1162/jocn.2009.21368 19925182

[B46] Raposo A. Mendes M. Marques J. F. (2012). The hierarchical organization of semantic memory: executive function in the processing of superordinate concepts. Neuroimage 59, 1870–1878 10.1016/j.neuroimage.2011.08.072 21906688

[B47] Sanjuan A. Forn C. Ventura-Campos N. Rodriguez-Pujadas A. Garcia-Porcar M. Belloch V. (2010). The sentence verification task: a reliable fmri protocol for mapping receptive language in individual subjects. Eur. Radiol. 20, 2432–2438 10.1007/s00330-010-1814-7 20467871

[B48] Shackman A. J. Salomons T. V. Slagter H. A. Fox A. S. Winter J. J. Davidson R. J. (2011). The integration of negative affect, pain and cognitive control in the cingulate cortex. Nat. Rev. Neurosci. 12, 154–167 10.1038/nrn2994 21331082 PMC3044650

[B49] Sheth S. A. Mian M. K. Patel S. R. Asaad W. F. Williams Z. M. Dougherty D. D. (2012). Human dorsal anterior cingulate cortex neurons mediate ongoing behavioural adaptation. Nature 488, 218–221 10.1038/nature11239 22722841 PMC3416924

[B50] Sul S. Choi I. Kang P. (2011). Cultural modulation of self-referential brain activity for personality traits and social identities. Soc. Neurosci. 7, 280–291 10.1080/17470919.2011.614001 21970690

[B51] Talairach J. Tournoux P. (1988). Co-Planar Stereotaxix Atlas of the Human Brain. Stuttgart: Thieme

[B52] van der Meer L. Costafreda S. Aleman A. David A. S. (2010). Self-reflection and the brain: a theoretical review and meta-analysis of neuroimaging studies with implications for schizophrenia. Neurosci. Biobehav. Rev. 34, 935–946 10.1016/j.neubiorev.2009.12.004 20015455

[B53] Vanderwal T. Hunyadi E. Grupe D. W. Connors C. M. Schultz R. T. (2008). Self, mother and abstract other: an fmri study of reflective social processing. Neuroimage 41, 1437–1446 10.1016/j.neuroimage.2008.03.058 18486489 PMC2559963

[B54] Volz K. G. Schubotz R. I. von Cramon D. Y. (2006). Decision-making and the frontal lobes. Curr. Opin. Neurol. 19, 401–406 10.1097/01.wco.0000236621.83872.71 16914980

[B55] Wechsler D. (1999). Wechsler Abbreviated Scale of Intelligence. San Antonio, TX: Pearson

